# HLA-B*57:01 allele prevalence in HIV-infected North American subjects and the impact of allele testing on the incidence of abacavir-associated hypersensitivity reaction in HLA-B*57:01-negative subjects

**DOI:** 10.1186/s12879-017-2331-y

**Published:** 2017-04-11

**Authors:** Catherine Butkus Small, David A. Margolis, Mark S. Shaefer, Lisa L. Ross

**Affiliations:** 1 Weill Cornell Medical College, New York, NY USA; 2 New York Medical College, Valhalla, NY USA; 3 ViiV Healthcare, Research Triangle Park, NC USA

**Keywords:** Abacavir, Lamivudine, Hypersensitivity, HLA-B*57:01, HIV infection

## Abstract

**Background:**

The presence of the HLA-B*57:01 allele in HIV-infected subjects is associated with a higher risk of abacavir-associated hypersensitivity reaction (ABC HSR). HLA-B*57:01 allele prevalence varies in different populations, but HLA-B*57:01 testing with immunological confirmation has had a negative predictive value for ABC HSR between 97 and 100%.

**Methods:**

In the ASSURE study (EPZ113734), the HLA-B*57:01 prevalence in virologically suppressed, antiretroviral treatment–experienced, HIV-infected subjects from the United States, including Puerto Rico, was assessed.

**Results:**

Three hundred eighty-five subjects were screened; 13 were HLA-B*57:01 positive and 372 were negative. Only HLA-B*57:01-negative, abacavir-naive subjects were eligible to enroll into the ASSURE trial. Eleven of the 13 subjects who possessed the HLA-B*57:01 allele were white, the other 2 were African-American. There was no geographic clustering of HLA-B*57:01-positive subjects, and the incidence correlated roughly with those states with the greatest numbers of subjects screened. Similarly, there was no statistically significant correlation between subjects who possessed or lacked the allele and age, gender, ethnicity or CD4+ T-cell numbers. The incidence of ABC HSR following abacavir initiation was also evaluated. Only 1 of 199 HLA-B*57:01-negative subjects (an African-American male) randomized to receive abacavir-containing treatment developed symptoms consistent with suspected ABC HSR; ABC HSR was not immunologically confirmed.

**Conclusions:**

These findings confirm the utility of HLA-B*57:01 allele testing to reduce the frequency of ABC HSR. The prevalence of HLA-B*57:01 positivity was higher in white than in African-American subjects. In HLA-B*57:01-negative subjects, suspected ABC HSR is very rare, but should lead to discontinuation of abacavir when ABC HSR cannot be definitively excluded from the differential diagnosis.

**Trial registration:**

The ASSURE (EPZ113734) study was registered on ClinicalTrials.gov registration on April 8th 2010 and the registration number is NCT01102972.

## Background

The HLA-B*57:01 allele is associated with a higher risk of abacavir-associated hypersensitivity reaction (ABC HSR). This association resulted in a change in treatment strategies, and independent guidelines now endorse HLA-B*57:01 screening in HIV-positive patients prior to initiating ABC-containing regimens [1–3]. Before this association was determined, ABC HSR rates reported from clinical studies in which adult HIV-positive subjects were not prospectively tested for the HLA-B*57:01 allele before receiving ABC-containing treatment ranged from 3 to 6% [4–8]. A review article identified nine cohort or clinical studies where abacavir was used in HIV-1 infected pediatric patients (only one study included prospective HLA-B*57:01 at screening), and the pooled incidence rate of ABC HSR was 2.2% [9]. Several studies conducted after the association was hypothesized investigated the positive and negative predictive values of HLA-B*57:01 testing with immunological confirmation by patch testing. In the Western Australian HIV Cohort study, testing for the presence of the HLA-B*57:01, HLA-DR7 and HLA-DQ3 alleles had a positive predictive value for hypersensitivity of 100 and a negative predictive value of 97% [10], while in the randomized PREDICT-1 study, the presence of the HLA-B*57:01 allele was associated with a positive predictive value of 47.9% and a negative predictive value of 100% for immunologically confirmed ABC HSR [5]. In the US-based SHAPE study, the positive and negative predictive values of HLA-B*57:01 testing were estimated to be 50 and 100%, respectively [6]. In a smaller study conducted in Spain in which immunological confirmation was not performed, the positive and negative predictive values were 92 and 63%, respectively [8].

In the first randomized clinical study in the United States to prospectively screen and enroll only HLA-B*57:01-negative subjects (ARIES), there was a reported drop in the incidence of suspected ABC HSR to 0.8% [11]. The current study was the second randomized and controlled US study to prospectively screen and enroll only HLA-B*57:01-negative subjects. It has been reported in subsequent studies in which dolutegravir (DTG) has been used, either in combination with the fixed-dose combination (FDC) of ABC/lamivudine (3TC) or the FDC of DTG/ABC/3TC, that hypersensitivity events were observed in less than 1% of treated subjects. However, it was not possible to determine clinically whether the hypersensitivity reaction was caused by ABC or DTG [12].

The prevalence of this allele varies in different populations, with whites of European ancestry having higher frequencies of the HLA-B*57:01 allele than other racial or ethnic groups, ranging from 5 to 8% [6, 10, 13–16]. In the United States, there is a lower frequency of the HLA-B*57:01 allele in African Americans, with a reported frequency between 2.3 and 4% [14, 16].

To further investigate the prevalence of the HLA-B*57:01 allele in the United States and the impact of prospective HLA-B*57:01 allele testing on reducing the incidence of suspected ABC HSR, these factors were assessed in HIV-infected, virologically suppressed, antiretroviral treatment (ART)–experienced subjects from the United States (including Puerto Rico) who sought to enroll in the ASSURE study.

## Methods

### Study design

The ASSURE study (EPZ113734) was an open-label, multicenter, non-inferiority study in HIV-infected, ART-experienced subjects. Additional information on the inclusion/exclusion criteria, including a link to the study protocol, is available in a prior publication [17]. Briefly, HIV-infected subjects from centers in mainland United States and Puerto Rico were eligible for enrollment if they were ≥18 years of age, had a confirmed HIV RNA ≤75 copies/ml at screening, and had been on a regimen of tenofovir/emtricitabine (TDF/FTC; 200-mg/300-mg fixed-dose tablet; Gilead Sciences, Foster City, California) and atazanavir/ritonavir (ATV/r; 300-mg tablet of atazanavir [Bristol Myers Squibb, New York, New York] + 100-mg tablet of ritonavir [Abbott, Abbott Park, Illinois]) for at least 6 months immediately prior to study enrollment. Subjects could have had up to 2 additional prior ART regimens as long as the regimen switch was not due to virologic failure. There were no CD4+ lymphocyte count restrictions. While a prior HIV genotype was not required, subjects were ineligible if a prior viral genotype contained resistance-associated mutations to any study medications. Subjects were excluded if they were positive for the HLA-B*57:01 allele or for hepatitis B surface antigen, required use of prohibited medications, had medical conditions that could compromise their safety or interfere with drug absorption, had protocol-specified abnormal laboratory values, or had a creatinine clearance of <50 mL/min. Enrolled subjects were randomized 2:1 to receive abacavir/lamivudine (ABC/3TC; 600/300 mg fixed-dose tablet, ViiV Healthcare, Research Triangle Park, North Carolina) and 400 mg ATV or to continue TDF/FTC and ATV/r. All subjects provided written informed consent. The study received approval by the ethics review boards at each of the participating centers and was conducted in accordance with Good Clinical Practice. All of the HLA-B*57:01 testing was performed at Quest Diagnostics (Chantilly, VA) using the LABType® SSO B kit (ONE LAMBDA, Canoga Park, CA), which combines Luminex technology with reverse sequence–specific oligonucleotide DNA typing.

### Statistical analysis

The prevalence of suspected ABC HSR was determined starting at Day 1 for subjects receiving ABC-containing ART. Descriptive statistics were primarily used for data presentation. The Wilcoxon rank sum test and Fisher’s exact test were used to evaluate study population differences.

## Results

HLA-B*57:01 testing was performed during screening on 385 HIV-infected subjects from the United States (including Puerto Rico). The demographics of the population are shown in Table 1. None of the variables were statistically significant (*P* < 0.05) between the HLA-B*57:01 positive and negative subjects. African-Americans comprised 35% of the 372 HLA-B*57:01-negative subjects in the screening population, while the remaining HLA-B*57:01-negative subjects included 58% white and 6% Asian subjects. The median CD4 cell count was somewhat lower for subjects who were HLA-B*57:01-positive (median 321 cells/mm^3^) compared with HLA-B*57:01-negative subjects (482 cells/mm^3^), however this difference was not statistically significant (*p* = 0.0695, Wilcoxin rank sum test).

**Table 1:** Demographic comparison of HIV-infected subjects who were positive or negative for the HLA-B*57:01 allele

HIV-1-Infected Subjects	Screening Population HLA-B*57:01 Positive (*n* = 13)	Screening Population HLA-B*57:01 Negative (*n* = 372)	Total Enrolled Population HLA-B*57:01 Negative (*N* = 296)
Median Age (range)	42 (26–62)	44 (19–70)	43.5 (20–68)
Male, *n* (%)	9 (69%)	298 (80%)	234 (79%)
Race			
African-American/African Heritage, *n* (%)	2 (15%)	132 (35%)	102 (34%)
White, *n* (%)	11 (85%)	216 (58%)	177 (60%)
Asian/Other, *n* (%)	0	24 (6%)	17 (6%)
Ethnicity			
Hispanic/Latino, *n* (%)	2 (15%)	88 (24%)	77 (26%)
Median CD4 cells/mm^3^ (range) based on Day 1 values	321 (129–984)	482 (19–1553)	489.5 (77–1479)^a^

^a^This difference was not statistically significant (*P* = 0.0695, Wilcoxon rank sum test)

Thirteen of the 385 screened subjects (3.4%) were positive for the HLA-B*57:01 allele. Eleven of these subjects (85%) were white, and the other 2 were African-American (15%). When categorized by ethnicity, 2 of the 13 patients who were positive for the HLA-B*57:01 allele were of Hispanic/Latino ethnic background. Due to the small sample size, the higher prevalence of the HLA-B*57:01 allele in white subjects was not statistically significant when compared to other races (*P* = 0.0827, 2-sided Fisher’s exact test).

**Fig. 1:**
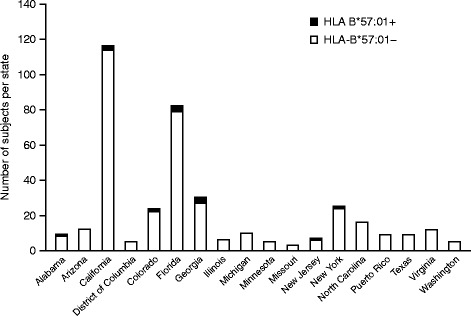
HLA-B*57:01 prevalence by state within the subject population at the screening visit

To examine whether the prevalence of HLA-B*57:01-positive subjects was increased in a particular geographic region, the HLA-B*57:01-positive prevalence was compared to the number of subjects screened within that area (Fig. 1). No geographic increase was identified, as 11 of the 13 HLA-B*57:01-positive subjects were from the 5 states with the greatest numbers of study subjects screened.

### Incidence of suspected ABC HSR in HLA-B*57:01-negative subjects

Two hundred ninety-six subjects met all study eligibility requirements and were enrolled. Based on the 2:1 study randomization, 199 subjects initiated ABC/3TC and ATV therapy, while 99 subjects remained on their pre-enrollment regimen of TDF/FTC and ATV/r.

Suspected ABC HSR is typically observed shortly after the initiation of ABC therapy; 93% of these reactions have been reported to occur during the first 6 weeks of treatment [4]. During the first 6 months of the ASSURE study, one of the 199 HLA-B*57:01-negative subjects randomized to the ABC/3TC + ATV arm developed a suspected ABC HSR. This subject was a 44-year-old HLA-B*57:01-negative African-American male who had met all of the inclusion criteria. One week after starting ABC/3TC + ATV, the subject developed a rash with welt-like lesions that increased after taking his antiretroviral medications, diarrhea, nausea, headache, and insomnia. No liver function test abnormalities or fever were observed. His signs and symptoms progressed with continued ART dosing. Concomitant medications included enalapril and dapsone, both of which he was taking for 1 year. At the time of the suspected ABC HSR, the subject had a non-reactive rapid plasma reagin (RPR) antigen for syphilis, a negative monospot test, and a negative test for cytomegalovirus IgM antibody. The HLA-B*57:01 test was repeated by the same diagnostic laboratory, and this result confirmed the prior negative result for the HLA-B*57:01 allele; however, an ABC skin patch test was not performed. ABC was discontinued and the prior ART was resumed (TDF/FTC and ATV/r). The subject’s signs and symptoms resolved within 24 h after ABC discontinuation. No other cases of suspected ABC HSR were reported for this study.

### Incidence of study withdrawal due to other adverse events in HLA-B*57:01-negative subjects

An additional 3 subjects who had been randomized to the ABC/3TC + ATV treatment group also withdrew during the first 8 weeks on treatment due to adverse events. These included 1 subject with urticaria, 1 with mood alteration, and 1 with abdominal pain thought to be related to ABC exposure by the investigator; none demonstrated signs or symptoms consistent with ABC HSR.

## Discussion

Prospective testing for the presence of the HLA-B*57:01 allele was performed for 385 subjects during the screening procedure for eligibility in the ASSURE Study. As previously noted, while the association between the HLA-B*57:01 allele and ABC HSR has been extensively examined, limited data are available from other large randomized clinical studies that prospectively tested for the presence of the HLA-B*57:01 allele prior to randomization and reported on the frequency of the allele and its subsequent impact on the incidence of ABC HSR. The presence of a positive HLA-B*57:01 haplotype was relatively uncommon in this US-based study, with a total prevalence of 3%, and did not appear to be geographically clustered but appeared to be more proportional to the total number of subjects screened within a particular state.

In the 13 HLA-B*57:01-positive subjects, the prevalence of HLA-B*57:01 positivity was higher in white subjects (although these numbers were too small to assess significance) at 4.8%, while the frequency of HLA-B*57:01 positivity was 1.49% for African-Americans. No Asian subjects tested positive for the HLA-B*57:01 allele in this study. These HLA-B*57:01-positive frequencies are similar to those reported by Cao et al., who observed allele frequencies of 4.15% in whites of European ancestry, 2.3% in African-Americans, and 0.97% in Asian subjects within the US population [14].

A mechanism of action for ABC HSR has been described in which ABC binds to the HLA-B*57:01 antigen-binding cleft, altering its shape and chemistry. This reaction modifies the peptide loading complex, generating an array of neopeptides that are antigenic in nature and drive the T-cell autoimmune responses and systemic toxicity associated with ABC HSR [16–21].

While these data clearly provide a mechanistic rationale for development of ABC HSR, it cannot be ruled out that in very rare instances additional immunologic or toxicological considerations may be relevant for a very small subset of subjects. In our study, a single instance of suspected ABC HSR was observed in an HLA-B*57:01- negative African-American subject. Although the genotypic test for the HLA-B*57:01 allele was repeated and confirmed, a patch test to provide immunological confirmation was not performed. A review of the literature identified 8 additional case reports of suspected ABC HSR or of symptoms considered by the investigator to be suspicious of HSR in HLA-B*57:01-negative subjects [22–26]. One United Kingdom single site cohort study evaluated outcomes for 739 HLA-B*57:01 testing results performed by a single diagnostic testing laboratory and reported that 4 subjects developed symptoms they considered suspicious of HSR despite a negative HLA-B*57:01 result; 2 of these subjects had ABC skin patch testing performed, and 1 was reported as positive [22]. Of the remaining 4 reported cases, only one subject had ABC skin patch testing performed with a negative result. A novel in vitro enzyme-linked immunosorbent spot (ELISPOT) assay has been described, which measures cellular in vitro responses to ABC and might be useful to further characterize suspected ABC HSR risk in peripheral blood mononuclear cells from subjects who lack the HLA-B*57:01 allele [27].

Based on the literature, reports of suspected ABC HSR are extremely rare in HLA-B*57:01-negative patients. ABC HSR remains a clinical diagnosis and a diagnosis of possible ABC HSR can be made even when plausible alternate clinical diagnoses exist. Rash alone without additional symptoms of a systemic process including fever, respiratory symptoms, gastrointestinal symptoms, malaise or myalgias, does not suggest the presence of underlying ABC HSR. However, patients who develop a rash after initiating ABC should be closely monitored for the onset of systemic symptoms. If ABC HSR is suspected, ABC should be immediately discontinued, with careful clinical follow-up of the patient [11, 28, 29]. Patients for whom ABC is stopped should be counseled not to take ABC-containing products in the future and should dispose of any remaining ABC-containing products.

## Conclusions

The prevalence of HLA-B*57:01 allele in this United States–based ASSURE study was 3.4%. Prospective HLA-B*57:01 allele testing with subsequent enrollment of exclusively HLA-B*57:01-negative subjects resulted in a 0.5% incidence of suspected ABC HSR. These data confirm the utility of HLA-B*57:01 allele testing and the value of prospective screening of patients in routine clinical practice in preventing ABC HSR and the associated potential for morbidity and mortality. In HLA-B*57:01-negative subjects, a diagnosis of suspected ABC HSR appears to be rare, and a possible mechanism remains to be elucidated. However, if ABC HSR is suspected, it should result in discontinuation of ABC.

## Abbreviations

ABC HSR: Abacavir hypersensitivity
ABC/3TC: Abacavir/lamivudine
ART: Antiretroviral therapy
ATV/r: Atazanavir/ritonavir
ELISPOT: Enzyme-linked immunosorbent spot
FDC: Fixed-dose combination
RPR: Rapid plasma reagin
TDF/FTC: Tenofovir/emtricitabine
